# Supplementary material for: Personalized Medicine in Infant Population with Cancer: Pharmacogenetic Pilot Study of Polymorphisms Related to Toxicity and Response to Chemotherapy
Source: Cancers (Basel). 2023 Feb 23;15(5):1424. doi: 10.3390/cancers15051424 (PMC10000841; doi:10.3390/cancers15051424)
Supplement: Supplementary file 1 [file cancers-15-01424-s001.zip › Table S2. Genotyping panel.pdf]

Table S2. SNPs panel. Information regarding drug agencies recommendation, drug labels and levels of evidence according to PharmGKB (accessed 2019). RNPGx: French National Network of Pharmacogenetics; EMA: European Medicines Agency; FDA: Food and Drug Administration; Sw: Swissmedic

SNP	Gene	Wild type	Variant	Guides	Drug label		Level of evidence	
					Agency	Status	Adult	Pediatric
**rs1042522**	*TP53*	C	G	NO	NO	X	3	X
**rs1045642**	*ABCB1*	G	A	NO	NO	X	3	3
**rs1051266**	*SLC19A1*	C	T	NO	NO	X	3	X
**rs11045879**	*SLC01B1*	C	T	RNPGx	NO	X	3	3
**rs112445441**	*KRAS*	G	A	NO	NO	X	3	X
**rs1127354**	*ITPA*	C	A	NO	NO	X	3	3
**rs1142345**	*TPMT*	T	C	NO	NO	X	1A	1A
**rs11615**	*ERCC1*	G	A	NO	NO	X	3	X
**rs116855232**	*NUDT15*	C	T	NO	EMA	Processable	1A	1A
**rs121434568**	*EGFR*	G	A	NO	FDA	Required	1A	X
**rs12248560**	*CYP2C19*	C	T	NO	NO	X	3	X
**rs1517114**	*C8orf34*	G	C	NO	NO	X	3	X
**rs16853826**	*ATIC*	G	A	NO	NO	X	3	X
**rs1695**	*GSTP1*	G	A	NO	NO	X	3	X
**rs1800460**	*TPMT*	C	T	NO	NO	X	1A	1A
**rs1800462**	*TPMT*	C	G	NO	NO	X	NO	X
**rs1800566**	*NQO1*	G	A	NO	NO	X	3	X
**rs1800584**	*TPMT*	C	T	NO	NO	X	NO	X
**rs1801019**	*UMPS*	G	C	NO	NO	X	NO	X
**rs1801131**	*MTHFR*	A	C	RNPGx	NO	X	3	3
**rs1801133**	*MTHFR*	A	G	RNPGx	NO	X	3	3
**rs1801158**	*DPYD*	G	A	NO	NO	X	1A	X
**rs1801160**	*DPYD*	G	A	NO	NO	X	1A	X
**rs1801274**	*FCGR2A*	A	G	NO	NO	X	3	X
**rs1801394**	*MTRR*	A	G	NO	NO	X	3	3
**rs2073618**	*TNFRSF11B*	G	C	NO	NO	X	3	X
**rs2228001**	*XPC*	T	G	NO	NO	X	3	X
**rs2234693**	*ESR*	T	C	NO	NO	X	3	X
**rs2299939**	*PTEN*	C	A	NO	NO	X	3	X
**rs246240**	*ABCC1*	A	G	NO	NO	X	3	X
**rs25487**	*XRCC1*	C	T	NO	NO	X	2B	X
**rs2612091**	*ENOSF1*	C	T	NO	NO	X	3	X
**rs2699887**	*PIK3CA*	C	T	NO	NO	X	3	X
**rs2740574**	*CYP3A4*	A	G	NO	NO	X	3	X
**rs316019**	*SLC22A2*	A	C	NO	NO	X	3	3
**rs3212986**	*ERCC1*	A	C	NO	NO	X	3	X
**rs3215400**	*CDA*	C	Del	NO	NO	X	3	3
**rs3740066**	*ABCC2*	T	C	NO	NO	X	3	X
**rs3768142**	*MTRR*	G	T	NO	NO	X	3	3
**rs3892097**	*CYP2D6*	C	T	NO	NO	X	1A	X
**rs3918290**	*DPYD*	C	T	RNPGx	NO	X	1A	X
**rs396991**	*FCGR3A*	A	C	NO	NO	X	3	X

**rs4124874**	*UGT1A1*	T	C	NO	NO	X	3	X
**rs4148323**	*UGT1A1*	G	A	NO	Sw	Informative	1B	X
**rs4149015**	*SLC01B1*	G	A	NO	NO	X	3	X
**rs4149056**	*SLC01B1*	C	T	NO	NO	X	3	3
**rs4244285**	*CYP2C19*	G	A	NO	NO	X	3	X
**rs4673993**	*ATIC*	C	T	NO	NO	X	2B	X
**rs4802101**	*CYP2B6*	T	C	NO	NO	X	3	X
**rs4880**	*SOD2*	A	G	NO	NO	X	3	X
**rs55886062**	*DPYD*	A	C	RNPGx	NO	X	1A	X
**rs602950**	*CDA*	A	G	NO	NO	X	3	X
**rs61886492**	*FOLH1*	G	A	NO	NO	X	3	3
**rs67376798**	*DPYD*	T	A	RNPGx	NO	X	1A	X
**rs683369**	*SLC22A1*	G	C	NO	NO	X	3	X
**rs716274**	*DYNC2H1*	A	G	NO	NO	X	3	X
**rs75017182**	*DPYD*	G	C	NO	NO	X	1A	X
**rs776746**	*CYP3A5*	A	G	NO	NO	X	1A	1A
**rs7779029**	*SEMA3C*	T	C	NO	NO	X	3	X
**rs8187710**	*ABCC2*	G	A	NO	NO	X	3	X
**rs924607**	*CEP72*	C	T	NO	NO	X	3	3
**rs2612091**	*ENOSF1*	C	T	NO	NO	X	3	X
**rs2699887**	*PIK3CA*	C	T	NO	NO	X	3	X
**rs2740574**	*CYP3A4*	A	G	NO	NO	X	3	X
**rs316019**	*SLC22A2*	A	C	NO	NO	X	3	3
**rs3212986**	*ERCC1*	A	C	NO	NO	X	3	X
**rs3215400**	*CDA*	C	Del	NO	NO	X	3	3
**rs3740066**	*ABCC2*	T	C	NO	NO	X	3	X
**rs3768142**	*MTRR*	G	T	NO	NO	X	3	3
**rs3892097**	*CYP2D6*	C	T	NO	NO	X	1A	X
**rs3918290**	*DPYD*	C	T	RNPGx	NO	X	1A	X
**rs396991**	*FCGR3A*	A	C	NO	NO	X	3	X
**rs4124874**	*UGT1A1*	T	C	NO	NO	X	3	X
**rs4148323**	*UGT1A1*	G	A	NO	Sw	Informative	1B	X
**rs4149015**	*SLC01B1*	G	A	NO	NO	X	3	X
**rs4149056**	*SLC01B1*	C	T	NO	NO	X	3	3
**rs4244285**	*CYP2C19*	G	A	NO	NO	X	3	X
**rs4673993**	*ATIC*	C	T	NO	NO	X	2B	X
**rs4802101**	*CYP2B6*	T	C	NO	NO	X	3	X
**rs4880**	*SOD2*	A	G	NO	NO	X	3	X
**rs55886062**	*DPYD*	A	C	RNPGx	NO	X	1A	X
**rs602950**	*CDA*	A	G	NO	NO	X	3	X
**rs61886492**	*FOLH1*	G	A	NO	NO	X	3	3
**rs67376798**	*DPYD*	T	A	RNPGx	NO	X	1A	X
**rs683369**	*SLC22A1*	G	C	NO	NO	X	3	X
**rs716274**	*DYNC2H1*	A	G	NO	NO	X	3	X
**rs75017182**	*DPYD*	G	C	NO	NO	X	1A	X
**rs776746**	*CYP3A5*	A	G	NO	NO	X	1A	1A
**rs7779029**	*SEMA3C*	T	C	NO	NO	X	3	X
**rs8187710**	*ABCC2*	G	A	NO	NO	X	3	X
**rs924607**	*CEP72*	C	T	NO	NO	X	3	3
