# Supplementary material for: Personalized Medicine in Infant Population with Cancer: Pharmacogenetic Pilot Study of Polymorphisms Related to Toxicity and Response to Chemotherapy
Source: Cancers (Basel). 2023 Feb 23;15(5):1424. doi: 10.3390/cancers15051424 (PMC10000841; doi:10.3390/cancers15051424)
Supplement: Supplementary file 1 [file cancers-15-01424-s001.zip › Table S3. Protocols.pdf]

Table S3: Protocols and therapeutic guidelines used in the treatment of study

LEUKEMIAS
- 2002 SEHOP PETHEMA ALL infants
- Recurrence LLA/SEHOP 2008
- Re-induction protocol relapsed or refractory AL; A Kolb, PG Steinherz
- Protocol LAL / SHOP-2005
- LAL SEHOP-PETHEMA 2013
- SEHOP-LANL 2001
- BFM-AML Relapse 2009. International Registry "Berlin-Frankfürt-Münster study group"
- LMA SHOP 2007

CENTRAL NERVOUS SYSTEM TUMORS
- SIOP LLG 2004 < 12kg. Children and Adolescents With Low Grade Glioma
- Protocol VINILO SIOPE: Phase-II study of vinblastine in combination with nilotinib in children, adolescents and Young adults with refractory or recurrent low-grade-glioma
- HART for Metastatic Medulloblastoma
- EURO-INFANT-HGG-2008 < 10kg
- HeadStart COG TRIAL
- SIOP CNS GCT II < 10kg

NEUROBLASTOMA
- Trial LINES: European Low and Intermediate Risk Neuroblastoma Protocol
- INES 99: Infant Neuroblastoma European Study 1999
- HR-NBL1 COJEC. Rapid Cojec Study
- Trial TOTEM: Trial topotecan and temozolamide
- COG P9642 study
- SIOPEN/ITCC Phase II study: TOTEM
- CCLG Relapse/Refractory High-Risk Neuroblastoma

WILMS/ NEFROBLASTOMA
- SIOP 2001 treatment metastatic disease < 12 kg
- WILMS SIOP 2001 bilateral
- SIOP UMBRELLA 2001 high risk III < 12 kg
- SIOP UMBRELLA 2016

RETINOBLASTOMA
- Consensus SEHOP Mayo de 2011. VCR+Carbo // VCR+Carbo+Etop // VCR+CFM+Doxo

HEPATOBLASTOMA
- SIOPEL 06 2008 5-10kg (Ramdomisation CDDP)
- SIOPEL 06 2004 AR 5-10kg PLADO
- SIOPEL 04 2004 AR 5-10kg

SARCOMA
- Protocol EpSSG RMS2005. High risk group < 12kg
- EpSSG RMS2005. SECOND LINE treatment
- RMS Refractory. Clinical Trial ISRCTN66172474 (<12Kg)
- Intergroup Rhabdomyosarcoma Group (IRSG) III, IV
- European rhabdoid registry 2010, SNC, < 18m
- SIOP Stage IV Malignant Mesenchymal Tumors in Children
- International Society of Pediatrics Oncology studies MMT 84 and MMT 89
